# Executive function associated with sexual risk in young South African women: Findings from the HPTN 068 cohort

**DOI:** 10.1371/journal.pone.0195217

**Published:** 2018-04-02

**Authors:** Molly Rosenberg, Audrey Pettifor, Mihaela Duta, Nele Demeyere, Ryan G. Wagner, Amanda Selin, Catherine MacPhail, Oliver Laeyendecker, James P. Hughes, Alan Stein, Stephen Tollman, Kathleen Kahn

**Affiliations:** 1 Department of Epidemiology and Biostatistics, Indiana University School of Public Health-Bloomington, Bloomington, IN, United States of America; 2 Department of Epidemiology, University of North Carolina-Chapel Hill, Chapel Hill, NC, United States of America; 3 Carolina Population Center, University of North Carolina-Chapel Hill, Chapel Hill, NC, United States of America; 4 MRC/Wits Rural Public Health and Health Transitions Research Unit (Agincourt), School of Public Health, Faculty of Health Sciences, University of the Witwatersrand, Johannesburg, South Africa; 5 Department of Experimental Psychology, University of Oxford, Oxford, United Kingdom; 6 INDEPTH Network, Accra, Ghana; 7 Umeå Centre for Global Health Research, Division of Epidemiology and Global Health, Department of Public Health and Clinical Medicine, Umeå University, Umeå, Sweden; 8 School of Health and Society, University of Wollongong, Wollongong, New South Wales, Australia; 9 Wits Reproductive Health and HIV Institute, University of the Witwatersrand, Johannesburg, South Africa; 10 Laboratory of Immunoregulation, NIAID, NIH, Baltimore, MD, United States of America; 11 Department of Medicine, Johns Hopkins University, Baltimore, MD, United States of America; 12 Department of Biostatistics, University of Washington, Seattle, WA, United States of America; 13 Department of Psychiatry, University of Oxford, Oxford, United Kingdom; University of Pennsylvania, UNITED STATES

## Abstract

**Purpose:**

Heightened sexual risk in adolescence and young adulthood may be partially explained by deficits in executive functioning, the set of cognitive processes used to make reasoned decisions. However, the association between executive function and sexual risk is understudied among adolescent girls and young women, particularly in low- and middle-income countries.

**Methods:**

In a cohort of 853 young women age 18–25 in rural Mpumalanga province, South Africa, we evaluated executive function with three non-verbal cognitive tests: I. a rule-finding test, II. a trail-making test, and III. a figure drawing test. Using log-binomial regression models, we estimated the association between lower executive function test scores and indicators of sexual risk (unprotected sex acts, concurrent partnerships, transactional sex, and recent HSV-2 infection).

**Results:**

In general, young women with lower executive function scores reported higher frequencies of sexual risk outcomes, though associations tended to be small with wide confidence intervals. Testing in the lowest quintile of Test I was associated with more unprotected sex [aPR (95% CI): 1.4 (1.0, 1.8)]. Testing in the lowest quintile of Test II was associated with more concurrent relationships and transactional sex [aPR (95% CI): 1.6 (1.1, 2.5) and 1.7 (1.3, 2.4), respectively], and testing in the lowest four quintiles of Test III was associated with more concurrent relationships [aPR (95% CI): 1.7 (1.0, 2.7)].

**Conclusions:**

These results demonstrate an association between low executive function and sexual risk in South African young women. Future work should seek to understand the nature of this association and whether there is promise in developing interventions to enhance executive function to reduce sexual risk.

## Introduction

As adolescents transition from childhood to adulthood they enter a high-risk period for negative sexual health outcomes. Globally, four million adolescents are HIV+[1] and 40 million are infected with herpes simplex virus type 2 (HSV-2).[2] The burden of these sexually transmitted infections (STI) is disproportionately carried by adolescents and young adults in sub-Saharan Africa;[1, 2] in South Africa alone, adolescents age 15–24 face a 7% HIV prevalence,[3] and 10% of young women age 13–24 test HSV-2+.[4]

Sexual risk-taking in adolescence and young adulthood may be partially explained by deficits in executive functioning. Executive function typically refers to a family of related domains used in high-level decision-making, providing the cognitive control necessary to filter information and override sensation-seeking impulses.[5–7] Many of these executive function domains could predict sexual risk-taking either independently or together due to the complex requirements of sexual decision-making.[8, 9] As examples, working memory is an executive function that plays a central role in planning and cognitive control and is negatively correlated with impulsive behaviors.[10, 11] Task switching, another key executive function, is the ability to flexibly shift attention between cognitive tasks, and is a critical step in deciding the sub-tasks necessary to achieve goals in the presence of distractions.[12–14]

In older adolescents and young adults, executive functioning is typically fully developed; however, we may still expect to observe a relationship between executive function and sexual risk. General risk-taking tends to peak in young adulthood, compared to younger adolescence.[15] This risk-taking peak could be explained by a subset of individuals who exhibit executive function weakness early in childhood[16] and maintain this weakness through adolescence[17] and into young adulthood.[18] The risk-taking may be exacerbated in later adolescence and young adulthood when environmental and social contexts provide more risk opportunities at this developmental period.[15, 18]

Limited research suggests that adolescents and young adults with higher levels of executive functioning do tend to report safer sexual behaviors.[19–23] However, much of the prior research in this area has been conducted either in small samples using neuro-imaging to assess cognitive function, or in larger, population-based studies using IQ tests or school performance as proxies for cognitive function. An exception, a longitudinal study of American adolescents, found that weaker working memory was associated with increased sexual risk taking.[22, 23] Like this promising work, nearly all prior studies were conducted in high-income countries, primarily the United States. Adolescents and young adults in low- and middle-income countries, where STI risk is higher and where social norms and expectations of adolescent behavior can be different, have been understudied.

In South Africa, where this study was conducted, young women are at exceptionally high risk for negative sexual health outcomes like HIV and other STI infections. By the time they reach their 20s, between 20–30% are already HIV+, with even higher prevalence estimates for HSV-2.[3, 4, 24] A shared proximal cause of these negative sexual health outcomes is engagement in some form of risky sexual activity. STI risk for young South African women tends to rise with behaviors such as inconsistent condom use, multiple and/or concurrent sexual partnerships, and engagement in transactional sex.[3, 24, 25] Although these behaviors are not uncommon among South African young women (8% multiple partnerships, 50% inconsistent condom use, and 14% engagement in transactional sex[3, 25]), it should be noted that, on average, young people in the United States report higher prevalence of sexual risk behaviors compared to young people in South Africa.[26]

In this study, we assess executive function with a set of three non-verbal cognitive tests in a population-based sample of rural South African young women and link those scores to behavioral and biological sexual risk outcomes, motivated by the hypothesis that lower executive function performance is associated with heightened sexual risk.

## Methods

### Study setting and study population

We analyzed data collected in the HIV Prevention Trial Network study 068 (HPTN 068). HPTN 068 was a Phase III randomized controlled trial to investigate whether cash transfers, conditional on school attendance, influence HIV risk in young South African women.[27] The study population was sampled from and nested within the Agincourt Health and socio-Demographic Surveillance System (HDSS) site, located in the rural Agincourt subdistrict of Mpumalanga Province, South Africa. Since 1992 the Agincourt HDSS has maintained an annual census update collecting full community vital event data, including births, deaths, and migrations, and socio-demographic data on all people living within the study area.[28]

**Fig 1:**
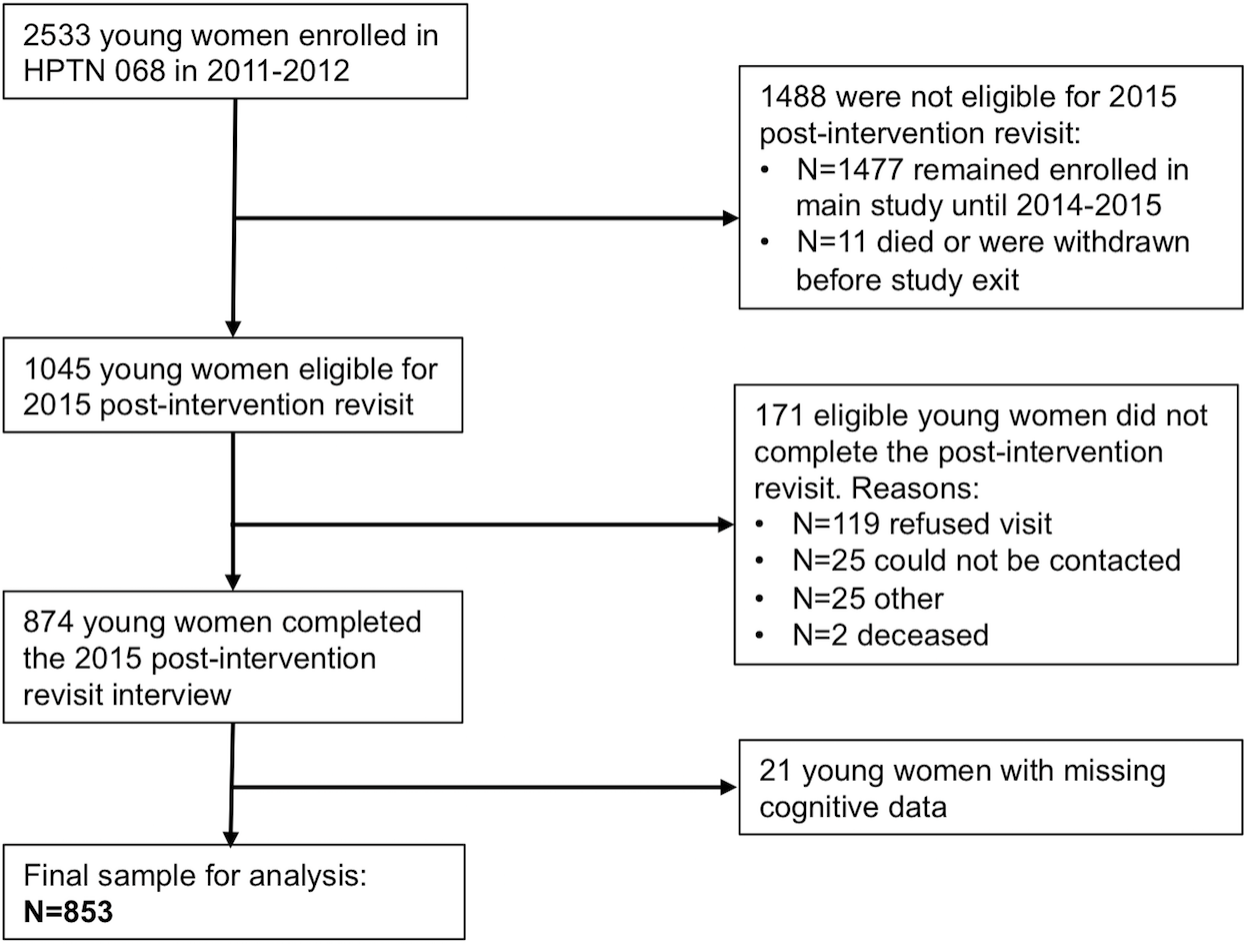
Flowchart of study sample construction.

In 2011–2012, 2533 young women, randomly sampled from within the broader AHDSS sampling frame, provided written informed consent (or written informed assent with parent/guardian written informed consent if under age 18 years) and enrolled in HPTN 068. Selection criteria for enrollment into the study included: age 13–20 years, current enrollment in grades 8, 9, 10, or 11, and not married or pregnant. School dropout, marriage, and pregnancy after enrollment did not preclude ongoing study participation. Study visits occurred at baseline and once annually for up to three years of follow-up. In 2015, HPTN 068 participants who had exited the study in 2012–2013 were invited to participate in an additional post-intervention visit, where cognitive data were collected for the first time. Overall, 853 young women completed the 2015 post-intervention revisit with complete cognitive data and were included in the analysis (Fig 1).

Ethical approval for the parent study was provided by the Office of Human Research Ethics at the University of North Carolina-Chapel Hill (#10–1868). Further ethical approval for the parent study was provided by the University of the Witwatersrand’s Human Research Ethics and the Mpumalanga Province Health Research and Ethics Committee. The Harvard Office of Research Administration and Indiana University Office of Research Compliance approved this secondary data analysis (#16–0359 and #1605028791, respectively).

### Data collection

In the 2015 visit, extensive socio-demographic and sexual behavior data were collected in an electronic survey conducted in the local Shangaan language. To minimize bias resulting from participants providing sensitive information directly to an interviewer, an ACASI (audio computer-assisted interviewing) component was incorporated into the survey for all sensitive modules. Participants provided cognitive data by filling in their responses on a hard-copy of a subset of the Oxford Cognitive Screen-Plus (OCS-Plus) (description below) alongside a trained fieldworker. At this and every previous study visit, blood was drawn for HIV and HSV-2 testing from those who had not previously tested positive for the respective test.

### Key measures

We assessed executive function with three sub-tests from the OCS-Plus screening tool, an innovative, domain-specific approach to cognitive assessment.[29] OCS-Plus relies on visual-oriented assessments with limited language demands, making it suitable for non-Western, low-literacy populations. It has been validated in a cohort of older adults living in the same rural community as the current study.[29] From the full battery of OCS-Plus sub-tests, we chose three sub-tests to administer to our study population of young women. These three sub-tests were designed to measure key domains of executive function, the cognitive area we theorized would be most relevant to the younger study population. A summary of the three tests are given below; detailed descriptions are previously published.[29]

1. *Rule-finding test*: To assess overall executive control (incorporating elements of task switching and working memory), participants were asked to track a red circle as it moved along three columns of differently shaded geometric shapes according to certain rules that would change without warning. Participants were instructed to predict where the circle would move next across a total of 52 moves and across five total rules, measuring the ability to make predictions based on observing patterns, and update the predictions when patterns change. The rule-finding score was calculated as the percent of total rules identified.
2. *Trail-making test*: To assess the cognitive flexibility to shift attention from one task to another (task switching), participants were presented with an arrangement of circles and squares of varying sizes. Participants were asked to draw lines connecting the squares in descending size order, and the circles in ascending size order, starting with the largest square and alternating between shape types. The trails score was calculated as the percent of correct connections, with baseline adjustment for the participant’s ability to connect one type of shape only.
3. *Figure drawing test*: To assess planning and non-verbal short-term memory, participants were first asked to copy a composite geometric figure consisting of 20 elements, then immediately asked to draw the figure from memory. Each drawing was individually scored, with each element receiving one point each for its presence, position, and accuracy, adding up to a maximum score of 3, and a 3-point penalty for including extra elements (maximum score of 60). The figure drawing test score was calculated as the score for the figure drawn from memory, with baseline adjustment for their ability to copy the figure directly.

We made coding decisions for each derived score separately using a data-driven approach. First, we visually inspected graphs of the prevalence of each sexual risk outcome across executive function score quintiles to derive information on the shape of the relationship or indication of threshold effect.

Based on this, we coded the rule-finding and trail-making tests with a dichotomous cutpoint separating the lowest quintile from all higher quintiles, and the figure drawing test with a dichotomous cutpoint separating the top quintile from all lower quintiles. Likelihood ratio tests supported the threshold decision-making around where to place the cutpoint; for each test, model fit did not significantly improve with the inclusion of indicators for each quintile (S1 Table).

As indicators of sexual risk, we analyzed three behavioral and one biological outcome:

1. *Unprotected sex*, defined by the self-report of at least one condom unprotected vaginal sex act in the last three months
2. *Concurrent relationships*, defined by the self-report of at least two of the three most recent partners as ‘ongoing’
3. *Transactional sex*, defined by the self-report of having received gifts or money from a sex partner and feeling obligated to have sex in return, among at least one of up to three sex partners in the last 12 months
4. *Recent HSV-2 infection*, defined as testing HSV-2+ at a study visit any time on or after January 1, 2013, among those who had not previously tested HSV-2+. This definition excludes prevalent baseline HSV-2 infections and incident HSV-2 infections prior to 2013 to minimize the temporal distance between infection and executive function tests. The median time between the last HSV-2 test and test was 1.6 years (IQR: 1.5–2.3 years). HSV-2 laboratory testing was conducted using Kalon™ HSV-2 gG2 ELISA (Kalon Biological, Ltd., Surrey, United Kingdom) [30].

Although HIV testing was also conducted at each wave of HPTN 068 data collection, we did not assess the relationship between cognitive function and recent HIV infection in this analysis due to the small number of infections since 2013 in the young women sampled (n = 27).

### Analysis

We estimated the association between each executive function test score and each sexual risk outcome using log-binomial regression models to yield prevalence ratios. To investigate whether the strength of the association varied with age, we tested for interaction between executive function test score and age, with categories separating the youngest group (ages 18 and 19) from the older group (ages 20–25). We estimated unadjusted as opposed to adjusted prevalence ratios in this exploratory analysis as the covariates we found to be significantly associated with executive function score (education and maternal education) are theoretically causal influences on executive function. Adjusting for a covariate at the beginning of a causal pathway that continues on to executive function and ultimately sexual risk may inappropriately diminish the observed association between executive function and sexual risk.

## Results

The young women ranged in age from 18 to 25 years, with median age 20 (interquartile range: 20–21) (Table 1). Nearly all (97%) reported never having been married. The study sample was relatively highly educated, with nearly three-quarters (74%) reporting a grade 12 or higher attainment and nearly half (43%) reporting ongoing education. Maternal education was low overall with less than one-quarter of young women (23%) reporting their mothers as high school graduates. Food insecurity in the last 12 months was reported by 24% of the young women.

**Table 1:** Socio-demographic and sexual risk characteristics of young women, by executive function test scores, rural South Africa, 2013–2015.

		Rule-finding test score			Trail-making test score			Figure drawing test score		
	Full sample N (%)	≤ 20^th^ percentile N (%)	> 20^th^ percentile N (%)	p	≤ 20^th^ percentile N (%)	> 20^th^ percentile N (%)	p	≤ 80^th^ percentile N (%)	> 80^th^ percentile N (%)	p
*Socio-demographic variables*										
**Age**				0.01			0.06			0.9
18–19	181 (21.2)	61 (19.9)	120 (21.9)		40 (28.4)	141 (19.8)		136 (21.5)	45 (20.6)	
20–21	485 (56.9)	161 (52.6)	324 (59.2)		70 (49.7)	415 (58.3)		360 (56.8)	125 (57.1)	
22–25	187 (21.9)	84 (27.5)	103 (18.8)		31 (22.0)	156 (21.9)		138 (21.8)	49 (22.4)	
**Marital status**				0.2			0.3			0.7
Never married	823 (97.4)	291 (96.4)	532 (98.0)		139 (98.6)	684 (97.2)		611 (97.3)	212 (97.7)	
Married	22 (2.6)	11 (3.6)	11 (2.0)		2 (1.4)	20 (2.8)		17 (2.7)	5 (2.3)	
Missing	8	4	4		0	8		6	2	
**Educational attainment**				0.1			0.09			0.04
< Grade 12	266 (31.5)	105 (34.8)	161 (29.7)		53 (37.6)	213 (30.3)		210 (33.5)	56 (25.8)	
≥ Grade 12	578 (68.5)	197 (65.2)	381 (70.3)		88 (62.4)	490 (69.7)		417 (66.5)	161 (74.2)	
Missing	9	4	5		0	9		7	2	
**Current school enrollment**				0.1			0.2			1.0
Yes	361 (42.7)	118 (39.1)	243 (44.8)		53 (37.6)	308 (43.8)		268 (42.7)	93 (42.9)	
No	484 (57.3)	184 (60.9)	300 (55.3)		88 (62.4)	396 (56.3)		360 (57.3)	124 (57.1)	
Missing	8	4	4		0	8		6	2	
**Maternal education**				0.02			0.07			0.2
Not HS graduate	600 (76.8)	230 (81.6)	370 (74.2)		111 (82.8)	489 (75.6)		452 (77.9)	148 (73.6)	
HS graduate	181 (23.2)	52 (18.4)	129 (25.9)		23 (17.2)	158 (24.4)		128 (22.1)	53 (26.4)	
Missing	72	24	48		7	65		54	18	
**Food insecurity (12 months)**				0.8			0.1			0.9
Yes	194 (23.6)	68 (23.1)	126 (23.8)		39 (28.5)	155 (22.6)		144 (23.5)	50 (23.8)	
No	629 (76.4)	226 (76.9)	403 (76.2)		98 (71.5)	531 (77.4)		469 (76.5)	160 (76.2)	
Missing	30	12	18		4	26		21	9	
*Sexual risk outcomes*										
**Ever sex**				0.8			0.6			0.1
Yes	582 (69.3)	205 (68.8)	377 (70.0)		95 (67.4)	487 (69.7)		423 (67.9)	159 (73.3)	
No	258 (30.7)	93 (31.2)	165 (30.4)		46 (32.6)	212 (30.3)		200 (32.1)	58 (26.7)	
Missing	13	8	5		0	13		11	2	
**Unprotected sex**^1^				0.01			0.6			0.1
Yes	174 (31.0)	75 (37.9)	99 (27.3)		30 (33.3)	144 (30.6)		134 (33.0)	40 (25.8)	
No	387 (69.0)	123 (62.1)	264 (72.7)		60 (66.6)	327 (69.4)		272 (67.0)	115 (74.2)	
Missing	21	7	14		5	16		17	4	
**Concurrent partners**^1^				0.7			0.03			0.03
Yes	92 (15.9)	34 (16.8)	58 (15.5)		22 (23.7)	70 (14.5)		75 (17.9)	17 (10.7)	
No	485 (84.1)	169 (83.3)	316 (84.5)		71 (76.3)	414 (85.5)		343 (82.1)	142 (89.3)	
Missing	5	2	3		2	3		5	0	
**Transactional sex**^1^^,^^2^				0.6			0.001			0.1
Yes	135 (26.1)	50 (27,6)	85 (25.3)		34 (40.5)	101 (23.3)		104 (28.1)	31 (21.1)	
No	382 (74.9)	131 (72.4)	251 (74.7)		50 (59.5)	332 (76.7)		266 (71.9)	116 (78.9)	
Missing	65	24	41		12	53		53	12	
**HSV-2 status**^**3**^				0.1			0.8			0.9
Positive	145 (17.0)	60 (19.6)	85 (15.6)		25 (17.7)	120 (16.9)		108 (17.1)	37 (16.9)	
Negative	707 (83.0)	246 (80.4)	461 (84.4)		116 (82.3)	591 (83.1)		525 (82.9)	182 (83.1)	
Missing	1	0	1		0	1		1	0	
**Recent HSV-2 infection**^**4**^				0.2			0.7			0.2
Positive	44 (6.8)	19 (8.5)	25 (5.9)		7 (6.1)	37 (7.0)		29 (6.1)	15 (9.0)	
Negative	601 (93.2)	204 (91.3)	397 (94.1)		108 (93.9)	493 (93.0)		449 (93.9)	152 (91.0)	
Missing	107	42	65		8	99		77	30	
*Correlations between test scores*		**Pearson correlation coefficient**		**p**	**Pearson correlation coefficient**		**p**	**Pearson correlation coefficient**		**p**
Rule-finding		1.00			0.08453		0.01	0.08865		0.01
Trail-making		0.08453		0.01	1.00			0.07672		0.03
Figure drawing		0.08865		0.01	0.07672		0.03	1.00		

^1^Outcome calculated among those who reported being sexually active (n = 582)

^2^Transactional sex was estimated among any of the last three sex partners reported by the young women with whom they were sexually active within the last 12 months

^3^HSV-2 infection identified at any of the study visits, including baseline among full study population

^4^HSV-2 infection identified at either of the last two study visits, among those who had not previously tested HSV-2+ (n = 752)

Significant socio-demographic differences by executive function test score were observed for educational attainment, maternal education, and age. Those with lower scores tended to report educational attainment below Grade 12, compared to those with higher scores. Similarly, young women with lower scores were less likely to report that their mothers were high school graduates compared to those with higher scores. Although the test scores were distributed differently across categorized age groups, the mean age in lower scorers compared to the higher scorers was qualitatively similar for the rule-finding test (20.5 versus 20.8), the trail-making test (20.6 versus 20.5), and the figure drawing test (20.6 vs. 20.6). In general, sexual activity and sexual risk outcomes were common (Table 1). Most of the young women (69%) reported sexual debut. Among those who reported sexual debut, nearly a third (31%) reported unprotected sex in the last three months, 16% reported concurrent relationships, and 26% reported transactional sex-based relationships. Over the course of the study (including baseline), 7% tested HIV+ and 17% tested HSV-2+. Among those who had not previously tested HSV-2+, 7% tested HSV-2+ at a study visit from 2013 onward. There were no significant differences in average age for young women who reported sexual risk outcomes compared to those who did not.

The three executive function tests were modestly correlated with each other with a positive relationship (Table 1). The Pearson correlation coefficients ranged from 0.077 to 0.089.

Several of the sexual risk outcomes we examined were more likely to occur in among those with lower executive function test scores than in those with higher scores (Table 2). For the rule-finding test, young women who scored in the lowest quintile were 40% more likely to report recent unprotected sex than those in higher quintiles [PR (95% CI): 1.4 (1.0, 1.8)]. For the trail-making test, young women who scored in the lowest quintile were 60% more likely to report concurrent relationships [PR (95% CI): 1.6 (1.1, 2.5)] and 70% more likely to report transactional sex-based relationships [PR (95% CI): 1.7 (1.3, 2.4)]. For the figure drawing test, young women who scored in the bottom four quintiles were 70% more likely to report concurrent relationships [PR (95% CI): 1.7 (1.0, 2.7)]. In general, the point estimates for most of the associations with each sexual risk outcome tended to be at or above the null, indicating that lower scorers tended to have higher levels of sexual risk, though confidence intervals often included the null.

**Table 2:** The association between low executive function and sexual risk outcomes, among 853 young women in rural South Africa, 2013–2015.

Outcome	Full sample PR (95% CI)	p	Age 18–19 PR (95% CI)	p	Age 20–25 PR (95% CI)	p	LRT X^2^	LRT p-value
*I*. *Rule-finding test*^*1*^								
Unprotected sex	1.4 (1.0, 1.8)	0.02	2.3 (1.2, 4.2)	0.007	1.2 (0.9, 1.6)	0.2	3.5588	0.06
Concurrency	1.1 (0.7, 1.6)	0.7	2.4 (1.0, 5.8)	0.06	0.9 (0.6, 1.4)	0.7	4.062	0.04
Transactional sex	1.1 (0.8, 1.5)	0.6	2.6 (1.3, 5.3)	0.009	0.9 (0.7, 1.3)	0.6	6.562	0.01
HSV-2	1.4 (0.8, 2.6)	0.2	2.7 (0.6, 11.7)	0.2	1.3 (0.7, 2.4)	0.5	0.9102	0.3
*II*. *Trail-making test*^*1*^								
Unprotected sex	1.1 (0.7, 1.5)	0.8	1.6 (0.8, 3.0)	0.2	0.9 (0.6, 1.4)	0.7	0.7352	0.4
Concurrency	1.6 (1.1, 2.5)	0.02	2.1 (0.9, 5.3)	0.1	1.5 (0.9, 2.5)	0.09	0.0178	0.9
Transactional sex	1.7 (1.3, 2.4)	0.0005	2.2 (1.1, 4.4)	0.03	1.7 (1.2, 2.4)	0.005	0.3304	0.6
HSV-2	0.9 (0.4, 1.9)	0.7	-		1.2 (0.5, 2.6)	0.7	-	-
*III*. *Figure drawing test*^*2*^								
Unprotected sex	1.2 (0.9, 1.6)	0.3	1.8 (0.8, 4.4)	0.2	1.1 (0.8, 1.5)	0.7	0.3602	0.6
Concurrency	1.7 (1.0, 2.7)	0.04	2.7 (0.6, 11,0)	0.2	1.5 (0.9, 2.6)	0.1	0.4148	0.5
Transactional sex	1.3 (0.9, 2.0)	0.1	2.4 (0.8, 7.3)	0.1	1.2 (0.8, 1.8)	0.3	0.8856	0.4
HSV-2	0.7 (0.4, 1.2)	0.2	0.8 (0.2, 4.1)	0.8	0.7 (0.3, 1.3)	0.2	0.0004	1.0

^1^Associations for the rule-finding and trail-making tests calculated comparing those who scored in the lowest test quintile to those in the highest four quintiles

^2^Associations for the figure drawing test calculated comparing those who scored in the lowest four test quintiles to those in the highest quintile

LRT = likelihood ratio test to examine whether the addition of an interaction term between an age indicator for age 18–19 and executive function test score improved model fit over a model without the interaction term. Smaller p-values indicate more improvement in model fit with addition of interaction term.

Trends in the age-stratified analyses differed by test (Table 2). For the rule-finding test, the associations between test score and sexual risk outcome tended to be isolated to and strongest in the youngest age category (ages 18–19). For example, in this age group, those who scored in the lowest rule-finding quintile were over two times as likely to report unprotected sex [PR (95% CI): 2.3 (1.2, 4.2)] and transactional sex [PR (95% CI): 2.6 (1.3, 5.3)].The point estimates for each outcome among those in the older age groups were much closer to the null with confidence intervals spanning the null. For the trail-making test and figure drawing test, the point estimates across age categories tended to be more stable with no dramatic age differences or trends. In general, across each test, the age-specific estimates were less precisely measured due to smaller sample sizes in each age category. Likelihood ratio tests generally confirmed that the addition of the interaction term between age and rule-finding test score improved model fit while the addition of the interaction term in the trails and figure drawing test models did not.

## Discussion

We found that lower executive function performance was associated with heightened sexual risk in a cohort of young women in rural South Africa. This trend was generally consistent across the three executive function tests and four behavioral and biological sexual risk outcomes we examined. Our findings are compatible with the guiding hypothesis that those with lower executive function are more likely to engage in riskier sexual behavior. Our results are also consistent with findings from prior work demonstrating the population-level association between adolescent cognitive competence and sexual behavior,[19–21] and working memory and sexual behavior,[22, 23] as well as with results from smaller, purposefully sampled, laboratory-based brain-mapping studies.[31, 32]

Compared to prior studies, our approach yields a fuller understanding of the relationship between executive function and sexual risk in adolescent girls and young women. First, the biological indicator of sexual risk we assessed (HSV-2 infection) provided an objective measure with which to compare the self-reported behavioral indicators. For the rule-finding test, the associations between executive function and HSV-2 were of similar directions and magnitudes compared to the associations with the behavioral outcomes, providing some reassurance that our rule-finding results are unlikely distorted by social desirability bias. However, the HSV-2 results were less similar to the behavioral results for the trail-making and figure drawing tests, raising the possibility of inaccurately measured behavioral outcomes. Second, the executive function assessments we used were designed to measure the specific executive function sub-domains of task switching, executive control, and short-term memory. Unlike studies that use gross measures of cognitive competency like academic achievement, we were able to isolate the more specific cognitive processes we hypothesized could influence sexual risk. Finally, we used a data-driven approach to make key decisions around the coding of executive function test scores, allowing us to be more confident that we were capturing the true shape of the relationship between each executive function domain and sexual risk in this population. Though useful in early studies where the relationship between variables cannot be accurately anticipated *a priori*, it should be noted that this data-driven approach can potentially overestimate the significance of effects of interest, so greater confidence in our results requires confirmation in future studies.

The associations across all three tests were generally small and of similar magnitude, though the statistically significant associations for each test were with different sexual risk outcomes. Although the three test scores were modestly correlated with each other, they were designed to measure separate domains of executive function, which could plausibly explain why they relate differently to different sexual risk outcomes. The trends we observed in the age-stratified analyses also differed by test, suggesting that the relative influence of some, but not all, executive function domains on sexual risk may change over the course of adolescence and young adulthood. The associations observed with the rule-finding test–a test designed to measure executive control—were strongest in the youngest age category (age 18–19). It is plausible that, as young women transition into adulthood and gain sexual experience, their sexual decision-making becomes less reliant on executive control as behavioral patterns develop and sexual decision-making requires less cognitive work. The associations observed with the trail-making test and figure drawing test did not differ across age groups, suggesting that task switching and planning processes may maintain associations with sexual risk outcomes into young adulthood. Our findings suggest that the relationship between executive function—with particular focus on executive control—and sexual risk in even younger age adolescents warrants further investigation.

Although sexual risk-taking in adolescence and young adulthood is often attributed to poor impulse regulation, it is also plausible that some aspects of sexual risk and sexual risk-taking involve calculated decisions that could require at least moderate executive function. For example, engaging in transactional sex can involve rational decisions that weigh perceived health costs against financial benefit,[33, 34] while engaging in unprotected sex can be viewed as rational when insisting on condom use would upend established social norms and gender norms.[35, 36] This countervailing mechanism provides a potential explanation for the relatively modest associations we observed in this study. This is particularly true in our study population of young adults between the ages of 18–25, many of whom may have reached an age when impulse regulation is already improving.

This study extends the scope of prior studies linking executive function to sexual risk in primarily high-income countries to now include young women in a non-Western setting with high background HIV prevalence. However, several factors limit the broader generalizability of our findings. First, the baseline selection criteria for HPTN 068 included only young women currently enrolled in school at baseline. The excluded young women may have had lower executive function as a cause or consequence of early school drop-out, skewing our sample towards higher executive function performance. This exclusion is particularly pertinent as teen pregnancy, an indicator of sexual risk-taking, is also both a cause and a consequence of school drop-out,[37] perhaps skewing our sample towards lower sexual risk-taking as well. Further, our study was limited to young women age 18–25 years. Investigating the relationship between executive function and sexual risk in young men and in younger adolescents should be a focus of future work. Finally, the three executive function tests we administered do not represent the full battery of cognitive assessments that might be relevant to healthy sexual decision-making. There is a need to expand the study to other cognitive measures, such as risk proclivity, impulsivity, and social cognition, to better understand how cognitive processes influence sexual risk. Impulsivity, in particular, has been established as an important mediating factor along the pathway from executive function and sexual risk.[23]

Due to the cross-sectional and observational nature of the data, cautious interpretation of the findings is warranted. The executive function tests were administered at a single point in time when the young women were aged 18–25 years. Given the unknown developmental trajectory of these young women with regards to executive function, these measures could potentially be representative of executive function performance throughout adolescence, or could reflect more advanced executive function than would prevail if measurements were taken at younger ages and earlier stages of cognitive development. Unlike the cognitive data, the sexual risk outcomes were assessed with reference to specific time periods: unprotected sex was measured with reference to the most recent three month period, concurrency was measured among ongoing current relationships, transactional sex was measured with reference to partnerships active in the last 12 months, and HSV-2 infections were restricted to incident infections at the last two study visits covering a time period of up to three years before the cognitive data were measured. Although we attempted to restrict the sexual risk outcomes to minimize the temporal distance from the executive function measures, it is possible that executive function processes could have developed since the sexual risk indicators occurred, which would wash out relationships that may have previously existed. Future work should focus on establishing the temporal relationship between executive function and sexual risk, which requires longitudinal data collected around the cognitive and sexual trajectories of adolescents and young adults.

If further evidence suggests that executive function is causally related to sexual risk outcomes, then there may be promise in devising new interventions to improve the sexual health of adolescents and young adults through improved executive function. Our data, in line with previous studies, suggest education as a promising target for improvements in executive function;[38, 39] nutrition[40–42], physical activity[43–46], and social protection[47] interventions also have potential. If interventions were able to yield long-term improvements in executive function, then we would expect improvements in reasoned decision-making. Through this mechanism we might expect to observe healthier sexual behaviors. However, to maximize any potential sexual health impact, an executive function intervention would almost certainly have to be combined with sexual health education and access to sexual health services to provide the supporting facts and resources upon which sexual decisions can be more effectively made.

## Supporting information

S1 Table Likelihood ratio tests indicate model fit is not significantly improved with the addition of covariates for each test score quintile. (DOCX)
