# Supplementary material for: Executive function associated with sexual risk in young South African women: Findings from the HPTN 068 cohort
Source: PLoS One. 2018 Apr 2;13(4):e0195217. doi: 10.1371/journal.pone.0195217 (PMC5880379; doi:10.1371/journal.pone.0195217)
Supplement: S1 Table — (DOCX) [file pone.0195217.s001.docx]

|  | -2 Log likelihood | |  |  |  |
| --- | --- | --- | --- | --- | --- |
| Outcome | Full model^1^ | Reduced model^2^ | Χ^2^ statistic | df | p-value |
| *Rule-finding test* |  |  |  |  |  |
| Unprotected sex | 843.925 | 841.1706 | 2.7544 | 3 | 0.4 |
| Concurrency | 579.449 | 577.7928 | 1.6562 | 3 | 0.7 |
| Transactional sex | 595.9812 | 595.1546 | 0.8266 | 3 | 0.8 |
| HSV-2 | 319.7122 | 316.0264 | 3.6858 | 3 | 0.3 |
| *Trails test* |  |  |  |  |  |
| Unprotected sex | 848.9816 | 848.9816 | 0 | 3 | 1.0 |
| Concurrency | 575.9118 | 575.9118 | 0 | 3 | 1.0 |
| Transactional sex | 586.681 | 586.681 | 0 | 3 | 1.0 |
| HSV-2 | 321.091 | 321.091 | 0 | 3 | 1.0 |
| *Figure drawing test* |  |  |  |  |  |
| Unprotected sex | 849.0508 | 848.935 | 0.1158 | 3 | 1.0 |
| Concurrency | 571.4502 | 569.7078 | 1.7424 | 3 | 0.6 |
| Transactional sex | 594.2938 | 592.4734 | 1.8204 | 3 | 0.6 |
| HSV-2 | 320.6016 | 320.1666 | 0.435 | 3 | 0.9 |

^1^Full model codes test scores with cutpoints at each of five quintiles

^2^Reduced model codes test scores with a single cutpoint separating the lowest quintile from the highest four quintiles
